# Extreme MetaboHealth scores in three cohort studies associate with plasma protein markers for inflammation and cholesterol transport

**DOI:** 10.1186/s12979-025-00527-7

**Published:** 2025-09-09

**Authors:** Daniele Bizzarri, Erik B. van den Akker, Marcel J. T. Reinders, René Pool, Marian Beekman, Nico Lakenberg, Nicolas Drouin, Kelly E. Stecker, Albert J. R. Heck, Edward F. Knol, Jeannette M. Vergeer, M. Arfan Ikram, Mohsen Ghanbari, Alain J. van Gool, Joris Deelen, BBMRI-NL, Dorret I. Boomsma, P. Eline Slagboom

**Affiliations:** 1 Department of Biomedical Data Sciences, Molecular Epidemiology, LUMC, Leiden, The Netherlands; 2 Department of Biomedical Data Sciences, Leiden Computational Biology Center, LUMC, Leiden, The Netherlands; 3 Delft Bioinformatics Lab, Delft University of Technology, Delft, The Netherlands; 4 Department of Biological Psychology, Vrije Universiteit Amsterdam, Amsterdam, The Netherlands; 5 Amsterdam Public Health Research Institute, Amsterdam, The Netherlands; 6 Leiden Academic Centre for Drug Research, Leiden University, Leiden, Netherlands; 7 Biomolecular Mass Spectrometry and Proteomics, Bijvoet Center for Biomolecular Research and Utrecht Institute for Pharmaceutical Sciences, Utrecht University, Utrecht, The Netherlands; 8 Netherlands Proteomics Center, Utrecht, The Netherlands; 9 Center of Translational Immunology and Dermatology/Allergology, University Medical Center Utrecht, Utrecht, The Netherlands; 10 Department of Epidemiology, Erasmus MC, Rotterdam, the Netherlands; 11 Translational Metabolic Laboratory, Department of Laboratory Medicine, Radboud University Medical Center, , Nijmegen, the Netherlands; 12 BBMRI-NL: https://www.bbmri.nl; see Consortium Banner Supplement S1, https://www.bbmri.nl; 13 Amsterdam Reproduction and Development (AR&D) Research Institute, Amsterdam, The Netherlands; 14 Dept of Complex Trait Genetics, Center for Neurogenomics and Cognitive Research, Amsterdam, , Vrije Universiteit Amsterdam, Amsterdam, The Netherlands; 15 Max Planck Institute for the Biology of Ageing, Cologne, Germany

## Abstract

The MetaboHealth score is an indicator of physiological frailty in middle aged and older individuals. The aim of the current study was to explore which molecular pathways co-vary with the MetaboHealth score. Using a Luminex cytokine assay and liquid chromatography-mass spectrometry-based proteomics we explored the plasma proteins associating with the difference in 100 extreme scoring individuals selected from two large population cohorts, the Leiden Longevity Study (LLS) and the Rotterdam Study (RS), and discordant monozygotic twin pairs from the Netherlands Twin Register (NTR). In addition, we estimated the heritability of the score using 726 monozygotic (MZ) and 450 dizygotic (DZ) twin pairs. In the contrasting extreme scoring individuals from LLS and RS, we uncovered significant differences in 3 (out of 15) cytokines (GDF15, IL6, and MIG), and 106 (out of 289) plasma proteins. The high, poor health related, score associated with 42 increased inflammatory and immune related protein levels (CRP, LBP, HPT) and lowered levels of 71 HDL remodeling and cholesterol transport related proteins (e.g. APOA1, APOA2, APOA4, and TETN). Using the NTR twins, we subsequently showed that the MetaboHealth score is moderately heritable (h^2^ = 0.4). In MZ twins selected for maximal discordance within a pair we found 68 serum proteins associated with the MetaboHealth score indicating that only a minor part of the associations observed in LLS and RS is likely explained by genetic influences. Taken together, our study sheds light on the intricate interplay between the MetaboHealth score, plasma proteins, cytokines, and genetic influences, paving the way for future investigations aimed at optimizing this mortality risk indicator.

**Supplementary Information:**

The online version contains supplementary material available at 10.1186/s12979-025-00527-7.

## Introduction

As the global human population rapidly ages, it is valuable to measure vulnerability and expected resilience of older individuals to support prevention and well-informed treatment aimed at enhancing well-being [1]. Efficient disease prevention hinges on possibilities for evaluating not only an individual’s disease risk, but also the overall physiological vulnerability in an early stage which is often referred to as biological age. Originally the biological age of individuals was estimated from a suite of physiological tests and biochemical clinical quantifications [2]. More recently explorations shifted towards comprehensive molecular (‘omics’) datasets, providing global information on an individual’s health status. Currently blood-based biomarkers to assess overall vulnerability in aging are constructed from molecular markers and based on chronological age, disease onset and mortality [3]. Here we focus on data from metabolomics and proteomics platforms representing such novel molecular markers.

Proton nuclear magnetic resonance (^1^HNMR) metabolomics enables a cost-effective and standardized assessment of a multitude of small circulating metabolites. Recent extensive collaborative efforts, like BBMRI-NL [4], FINSK/THL [5], COMETS [6], and the UK-Biobank [7], resulted in large datasets generated on the same Nightingale Health Plc. ^1^H-NMR metabolomics platform. This platform has been largely explored as a source for generating markers associated with a multitude of endpoints (e.g., type 2 diabetes [8], aging [4], risk factors [9], and disease onset [10]). It gained particular attention, after training the MetaboHealth score, using mortality as endpoint, in the largest study of its kind so far (44,000 individuals (from 12 different cohorts) and 5,500 incident deaths) [11]. This score stratifies mortality risk with a higher accuracy than conventional clinical variables, with lower and higher values indicating low and higher 5 years risk for mortality, respectively. The MetaboHealth score, though originally trained on mortality, predicts multiple conditions related to overall health decline associated with ageing, such as frailty [12], cognitive decline [13], cancer [14], in clinical studies as well as respiratory deficiencies [7]. In fact, MetaboHealth showed a high performance in predicting mortality and frailty when compared to nine existing biological age predictors generated from DNA methylation and metabolomics data based on chronological age (e.g. DNAmAge, MetaboAge) and mortality related phenotypes (e.g. GrimAge) [12]. The MetaboHealth score includes only 14 metabolic markers. Poor health, i.e. a high MetaboHealth score, is indicated by increased levels of total lipids in chylomicrons, extremely large VLDL, small HDL, histidine, leucine, valine, and albumin, a larger mean diameter for VLDL particles, and an increased ratio of polyunsaturated fatty acids to total fatty acids and decreased levels of glucose, lactate, isoleucine, phenylalanine, acetoacetate, and glycoprotein acetyls (GlycA). Although the MetaboHealth score offers an indication of physiological frailty, especially for older individuals, it remains largely illusive which pathophysiological mechanisms and corresponding blood factors are tracked by this mortality-trained risk score.

**Fig. 1:**
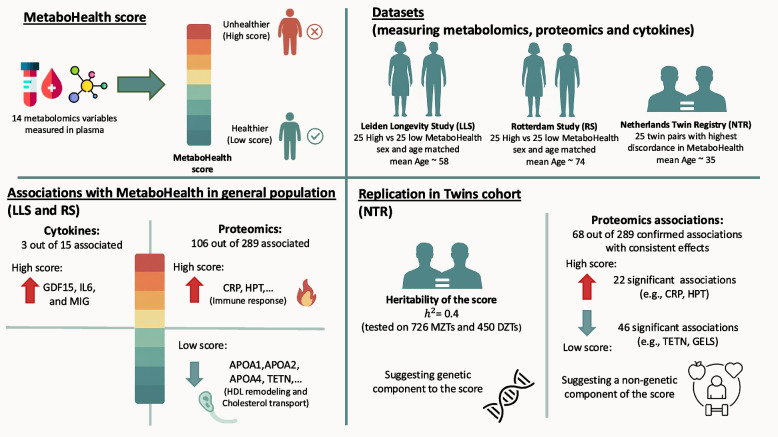
Graphical abstract summarizing the study design and main findings. The top panel provides an overview of the MetaboHealth framework (left) and the datasets used in this study (right). The bottom left panel outlines the primary analyses conducted in the RS and LLS-PAROFFS cohorts. The bottom right panel depicts replication and downstream analyses in the NTR cohort, showing that the MetaboHealth score is partially heritable and influenced by both genetic and non-genetic factors

The aim of the current study is, therefore, to explore which cytokines and molecular pathways represented in the plasma proteome co-vary with the MetaboHealth score. To this end, we performed profiling of cytokines and proteins in plasma of 50 out of 2200 Leiden Longevity Study (LLS) participants (mean age = 56 years) and 50 out of 2900 Rotterdam Study (RS) participants (mean age = 67 years) with the most extreme low and high values of the MetaboHealth score, and 50 monozygotic twins (MZTs) from the 25 most discordant MetaboHealth scoring pairs out of 2,754 twins in the Netherlands Twin Register (NTR) (mean age = 36 years) (Fig. 1). Considering that MZTs have identical genomes, their within-pair associations are free of genetic confounding [15]. Therefore, while the first two cohorts offer an insight into population associations, the MZTs differences inform to what extent MetaboHealth score differences in cytokines and plasma proteins are unconfounded by shared genetics and environment (Fig. 1). Overall, our exploration leads to a better understanding of the predictive power of the MetaboHealth score.

## Results

### Description of the dataset and study population

We performed a nested case–control study design [16], selecting 50 participants with extreme MetaboHealth scores, 25 with high score and 25 with low score, from the middle-aged cohort Leiden-Longevity-Study Partners-Offspring (LLS-PAROFFS, mean age = 56 years), and the Rotterdam Study (RS, mean age = 67 years) composed by older aged individuals (Figure S1B and Table 1). To minimize potential confounding, we ensured that the lower extreme samples were age- and sex-matched, with at least one high MetaboHealth score case (Figure S1A-B). Given that a high MetaboHealth score corresponds to higher mortality risk and poor health status, we categorized individuals with high scores as “cases” and the remainder as “controls”. These disparities in scores are manifested also in phenotypic characteristics. We observed that the cases show significantly higher BMI in LLS-PAROFFS (cases: 26.53 vs controls: 24.37 kg/m^2^) and a higher incidence of antihypertensive medication in RS (cases: 15 vs controls: 6 users) (Table 1). We observed in both study samples a lower level of lymphocyte percentage in the cases (RS: cases = 25.58% vs controls = 35.24%, LLS_PAROFFS: cases = 24.72% vs controls = 31.52%) (Fig. 1B). Interestingly, cases and controls shared similar phenotypic characteristics, despite being derived from two independent cohorts, differing for the significantly higher ages in RS (mean (age) _RS_ = 74 years, mean (age) _LLS-PAROFFS_ = 59 years), accompanied by a slightly elevated BMI in RS (mean (BMI) _RS_ = 25.86, mean (BMI) _LLS-PAROFFS_ = 24.86) and most importantly the larger MetaboHealth score contrast in RS (mean (MetaboHealth score contrast) _RS_ = 2.4, mean (MetaboHealth score contrast) _LLS-PAROFFS_ = 1.7) (Figure S1B, Table 1).

**Table 1:** Phenotypic characteristics of the selected MetaboHealth extremes

	**LLS-PARTOFFS**			**RS**			**NTR**		
Characteristic	**Control**	**Cases**	*p*-val	**Control**	**Cases**	*p*-val	**Control**	**Cases**	*p*-val
**Sex**			> 0.9			> 0.9			> 0.9
**Female**	13 (52%)	13 (52%)		12 (48%)	12 (48%)		18 (72%)	18 (72%)	
**Male**	12 (48%)	12 (48%)		13 (52%)	13 (52%)		7 (28%)	7 (28%)	
**Age**	58.64 (55, 65)	58.64 (55, 65)	> 0.9	74.04 (71, 77)	74.04 (71, 77)		35.48 (28, 39)	35.6 (28, 39)	> 0.9
**MetaboHealth**	−0.59 (−0.72,−0,47)	1.12 (0.96, 1.22)	< 0.001	−0.8 (−0.93, −0.67)	1.6 (1.34, 1.76)	< 0.001	0.71 (0.56, 1)	−0.47 (−0.61, −0.3)	< 0.001
**BMI**	23.25 (21.67, 25.03)	26.48 (22.72, 28.55)	0.045	25.17 (22.94, 27.53)	26.57 (24.94, 28.7	0.2	24.7 (21.63, 26.91)	24.41 (22.28, 26.31)	
**(Missing)**	7	8		0	1		0	0	
**Lipid medication**	1 (45%)	5 (23%)	0.2	4 (16%)	6 (26%)	0.5	25 (100%)	25 (100%)	
**(Missing)**	3	3		0	2		0	0	
**Blood_pres_lowering_med**			0.5			0.008			> 0.9
**FALSE**	12 (75%)			19 (76%)	8 (35%)		23 (92%)	24 (96%)	
**TRUE**	4 (25%)			6 (24%)	15 (65%)		2 (8%)	1 (4%)	
**(Missing)**	9	10		0	2		0	0	
**Monocyte %**	5.72 (5,6)	6.6 (4, 6)	0.5	6.72 (5, 8)	6.25 (5, 8)	0.5	9.74 (7.3, 11.1)	8.64 (7.8, 9.4)	0.2
**(Missing)**	0	0		0	1		2	0	
**Lymphocyte %**	31.52 (28.00, 34.00)	24.72 (19.00,29.00	0.001			0.005	31.67 (23.35, 36.6)	38.64 (34.2, 43.9)	0.03
**(Missing)**	0	0		0	1		2	0	

To investigate to what extent associations between proteins and MetaboHealth scores reflect confounding by shared genetic or environmental factors, we investigated the contrast in MetaboHealth score discordant MZTs from the Netherlands Twin Register dataset (NTR, mean age = 36 years) (Figure S1A). 25 Twin pairs with the highest discordance with respect to their MetaboHealth score were selected from 2,754 NTR participants. In accordance with the observations in RS and LLS, the individuals with higher MetaboHealth scores exhibited a significant reduction in the lymphocyte percentage (cases: 31.37% vs controls: 38.64%). Nonetheless, the NTR shows some relevant characteristics when compared to the other two studies. It represents the youngest population, with a 20-year gap (mean(age)_NTR_ = 36 years), it has a larger presence of females (36 out of 50), for which the MetaboHealth score previously showed a reduced accuracy [17]. These differences, and the selection criterion based on the twin discordancy, possibly lead to a diminished contrast in MetaboHealth score values (mean (MetaboHealth score contrast) _NTR_ = 1.18) (Figure S1B).

### Luminex cytokine assays: higher levels of GDF15, IL6 and MIG in the participants with a high MetaboHealth score

**Fig. 2:**
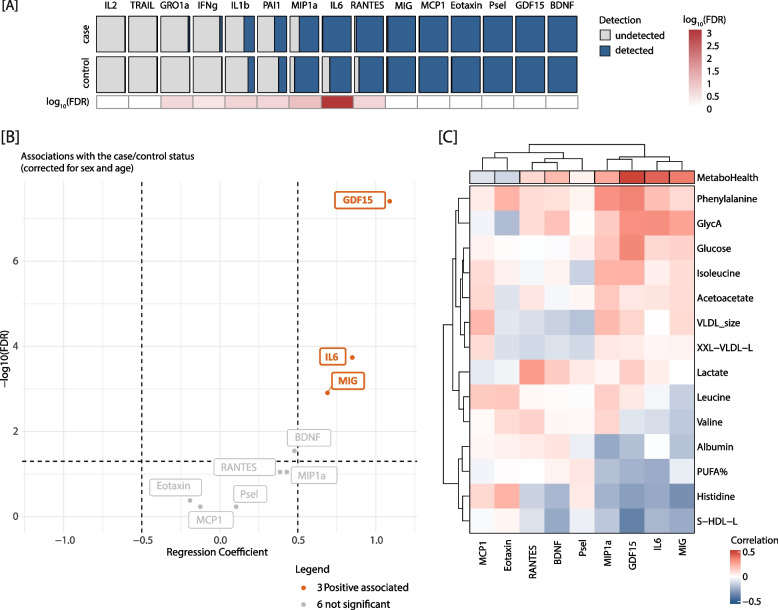
Increase GDF15, IL6, and MIG associate with MetaboHealth levels. **A** Differential detectability between cases (top) and controls (bottom) for the Luminex cytokine measures, with detected values in blue and undetected in grey. The heatmap on the bottom details the adjusted *p*-value of the Fisher test evaluating the significances of the differential detectability. **C** Volcano-plot showing the univariate associations between the cytokines levels and the participants’ case/control status corrected for sex, and age. In red the positively associated cytokines, in grey the ones not significant. **D** Heatmap depicting the correlations between each cytokine with the components of the MetaboHealth score

To explore the inflammatory state variation between the MetaboHealth score extremes, we quantified 15 cytokines on the Luminex platform (Materials and Methods for a complete explanation). Six out of the 15 (40%) cytokines (IL2, TRAIL, GRO1a, IFNg, ILB, and PAI1) were mostly undetectable, while the levels of the other cytokines were in the normal range (Figs. 2A and S2, and Supplementary Table S1). For instance, the highest IL-6 level is 35.42 pg/mL, which was observed in one of the cases in the RS cohort (Table S1), but this is still within the clinically accepted reference range [18]. This implies that most participants did not show any strong sign of inflammation at the time of sampling. However, on average, more cytokines were undetected in low MetaboHealth score participants across the LLS and RS cohorts, hinting at their overall lower inflammation rate (Fig. 2A). This discrepancy in detectability attained statistical significance in the case of IL6 (*p*-value = 0.001) (Fig. 2A). The same distinctive patterns between the high and low MetaboHealth score cohorts can be observed when considering the two cohorts separately (Figure S2B-C).

Our subsequent analyses focused on nine cytokines (MIP1a, IL6, RANTES, MIG, MCP1, Eotaxin, Psel, GDF15, and BDNF) exhibiting the fewest detectability issues (Fig. 2A). We compared cytokine levels between MetaboHealth score groups using linear models adjusted for age and sex. Interestingly, significantly higher levels of GDF15 (estimate = 1.08, *p* = 4.3 × 10^–9^, CI: [0.73:1.45]), IL6 (estimate = 1.05, *p* = 4.1 × 10^–5^, CI: [0.44:1.25]), and MIG (estimate = 0.95, *p* = 4.1 × 10^–4^ CI: [0.31:1.07]) were observed in the high MetaboHealth score group when considering LLS-PAROFFS and RS together (Fig. 2B). Adjusting for medication use (blood pressure lowering and statins) and cell counts (particularly lymphocyte %) mostly influenced the association with GDF15 (Figure S3A-D), yet it remained significant. Finally, reproducing the univariate associations separately for the two cohorts shows similar patterns (Figure S3F), underpinning their robustness.

To further investigate the origin of the observed signal, we looked into the correlation structure between cytokines. Cytokines were mostly independent of each other, showing only modest correlations (Figure S3E).Next, we estimated the correlation of the cytokines with the metabolomics components of the MetaboHealth score (Fig. 2C). While the MetaboHealth score exhibits the highest correlations with the cytokines, we also observed several other noteworthy correlations, i.e. strong positive correlations between GlycA, a metabolomics inflammation marker, and GDF15 (*r* = 0.33, *p* = 8.2 × 10^–4^), MIG (*r* = 0.27, *p* = 0.008), and IL6 (*r* = 0.33, *p* = 0.002). Intriguingly, GDF15 displayed also elevated positive correlations with glucose (*r* = 0.35, *p* = 4.2 × 10^–4^), phenylalanine (*r* = 0.35, *p* = 0.003), and isoleucine (*r* = 0.23, *p* = 0.021). In contrast, we uncovered prominent negative correlations between GDF15 and S-HDL-L (total lipids in small HDL) (*r* = −0.38, *p* = 9.9 × 10^–5^), and between MIG and Histidine (*r* = −0.33, *p* = 8.7 × 10^–4^).

### Plasma proteomics associated with extremes in MetaboHealth are enriched for inflammatory response and cholesterol transport pathways

**Fig. 3:**
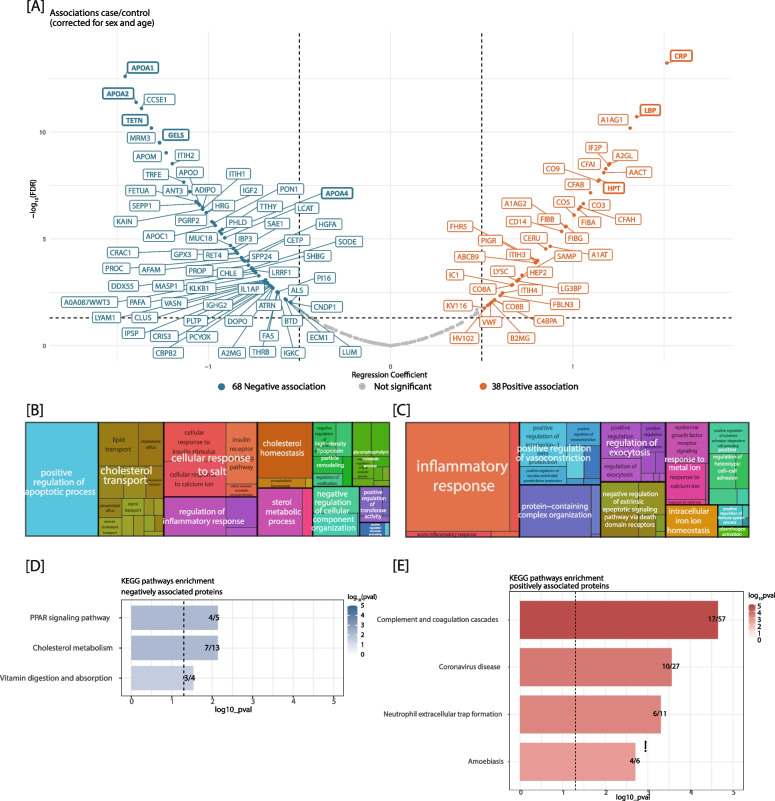
Quantitative plasma proteomics reveals proteins that associate with MetaboHealth scores in RS and LLS-PAROFFS cohorts. **A** Volcano plot depicting associations of proteins with MetaboHealth scores (corrected for sex, and age). In blue the negatively and in red the positively associated plasma proteins are depicted. Grouped significantly enriched Gene Ontology Biological Processes are shown for (**B**) the negatively and (**C**) positively associated plasma proteins. **D** and **E** depict the enriched KEGG pathways for the plasma proteins respectively negatively and positively associated with the MetaboHealth score

In the same two cohort samples (50 cases and 50 controls), we investigated plasma proteome profiles by a DIA-based quantitative plasma proteomics pipeline [19, 20] (Materials and Methods). 261 out of 337 measured plasma proteins (77%) passed the detection and quality control criteria (detailed in Materials and Methods). We identified 106 (68 negative and 38 positive) proteins significantly associated with MetaboHealth score extremes, after adjusting for age, and sex (Fig. 3A). APOA1 (estimate = −1.46, fdr = 2.44 × 10^–13^ CI: [1.8:−1.11]), APOA2 (estimate = −1.4, fdr = 4.02 × 10^–12^ CI: [−1.74:−1.05]), TETN (estimate = −1.31, fdr = 6.42 × 10^–11^ CI: [−1.67:−0.96]), GELS (estimate = −1.26, fdr = 3.18 × 10^–10^ CI: [−1.62:−0.91]), and APOA4 (estimate = −0.93, fdr = 5.71 × 10^–6^ CI: [−1.29:−0.56]) were the strongest negative associated proteins. In contrast, the positive acute phase proteins CRP (estimate = 1.51, fdr = 5.81 × 10^–14^ CI: [1.17:1.86]), LBP (estimate = 1.35, *p* = 1.88 × 10^–11^, CI: [0.99:1.7]), HPT (estimate = 1.14, p = 1.74 × 10^–8^, CI: [0.78:1.5]), were the strongest positive associated proteins. In line with what was observed for the cytokines, the additional correction for medication use had minimal impact on the univariate associations, while lymphocyte percentage exhibited a slightly more pronounced influence (Figure S4B-D). Furthermore, a meta-analysis to evaluate the associations separately in the two cohorts revealed similar results, with generally stronger signals in the RS (Figure S6A-B).

Subsequently, we explored the statistical interrelations and biological functionalities of the 106 plasma proteome features that exhibited significant association with the MetaboHealth score extremes. These proteins showed high correlations with GlycA, comparable to those observed for the MetaboHealth score (Figure S7A). Moreover, within the group of positively associated proteins, smaller clusters of highly correlated proteins are found (Figure S7B). To add biological interpretation, we employed KEGG and Gene Ontology to perform functional enrichments separately for the positively and negatively associated proteins (Figure S8). As expected, considering that lower MetaboHealth score values are related to healthier metabolic profiles, the negatively associated proteins demonstrated high enrichments for processes relating to “cholesterol transport”, “cholesterol metabolism”, and “high-density lipoproteins particle remodeling” (Figs. 3B, S8C and S8E). Conversely, the positively associated proteins were more enriched for “inflammatory response”, “complement and coagulation cascades”, and intriguingly, “coronavirus disease” (Fig. 3B, S8D and S8F). The latter can be interpreted as a validation, since about a dozen of inflammation related features, measured with the same proteomics platform, were previously linked to COVID19 outcomes [20]. Four of these twelve COVID19 related plasma proteins exhibited consistent statistically significant differences between the extremes of the MetaboHealth score in RS and LLS-PAROFFS (Figure S9).

### Genetic influences on the MetaboHealth score and analysis of plasma proteins in extreme discordant MZ twins

The NTR collected 726 complete MZTs and 450 dizygotic twins (DZTs) twin pairs with metabolomics data. We estimated the resemblance in MetaboHealth score as a function of zygosity in these pairs. The correlation between MZ twin pairs was *r* = 0.432 (95% CI = 0.370–0.489), while the correlation in DZTs was *r* = 0.230 (95% CI: [0.141–0.316]), indicating the MetaboHealth score is a moderately heritable trait (h^2^ = 0.4) (detailed information in Materials and Methods) [21].

**Fig. 4:**
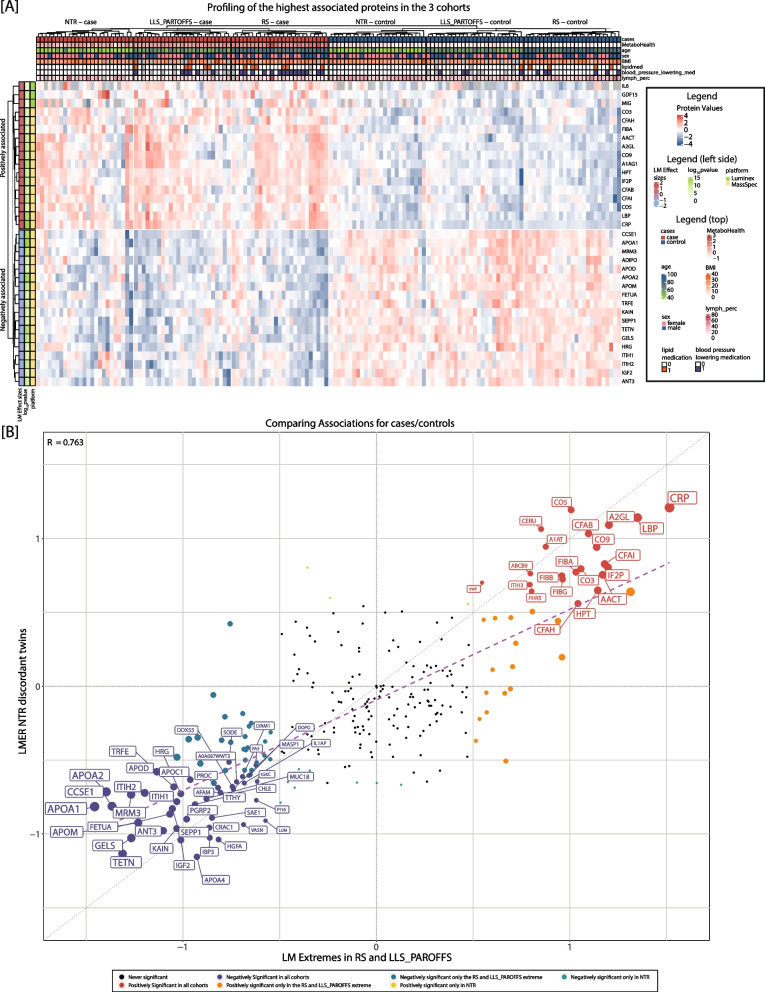
Plasma proteins associated with extreme MetaboHealth scores validated in the NTR dataset. **A** Profile depicting the values of the most significantly associated proteins (|estimate|> 1) and cytokines (y-axis) in all samples (x-axis), clearly separating the cases and controls in all 3 datasets. The annotation on the top show the phenotypic characteristics of all individuals. The annotation on the left shows the associations’ estimate, and log10(FDR) observed in RS and LLS, and the platform for each feature (Mass Spectrometry based Plasma Proteomics or Luminex). **B** Beta-beta plot comparing the linear models in the extremes of RS and LLS_PAROFFs on the x-axis and the linear mixed models in the NTR on the y-axis. Each dot corresponds to a feature, and it’s colored based on significance. The labels are shown only when the features are consistently significant in the 2 analyses

To exclude potential confounding from genetic factors within our associations, we conducted an MZTs discordant twin pairs design; comparing high and low scoring MetaboHealth genetically matched twins. This approach ensures that observed differences are not due to genetic factors. Therefore, we selected the subsample of 25 most discordant MZ twin pairs to further explore associations of the score to both cytokines and proteins. Consistent with the previous sections, the protein markers with the strongest associations with the MetaboHealth score also show a clear separation between cases and controls in the NTR dataset, albeit to a lesser degree than in the LLS and RS (Figs. 4, and S11). To take advantage of the genetic similarity of the MZTs, we tested for associated proteins using a linear mixed model (Methods). The cytokines did not show significant differences in this within-pairs design, although we observed similar trends to the results in LLS and RS, with elevated cytokines in twins with high MetaboHealth scores (Figure S11A). The proteomics analyses revealed a robust signal, identifying a total of 86 significant associations (Figure S11B). Notably, CRP (estimate = 1.19, fdr = 3.75 × 10^–5^ CI: [0.86:1.52]) and LBP (estimate = 1.15, fdr = 8.39 × 10^–5^ CI: [0.78:1.51]) once again emerged as the most prominently positively associated proteins, while TETN (estimate = −1.2, fdr = 9.69 × 10^–5^ CI: [−1.61:−0.82]) and GELS (estimate = −1.11, fdr = 4.43 × 10^–5^ CI: [−1.44:−0.79]) were confirmed as the most negatively associated proteins. Moreover, the weaker link between APOA1/2 and the MetaboHealth score in twins suggests that their associations in the other cohorts (LLS and RS) may be (partly) influenced by genetics. A correction for health factors had similar results as for the other two cohorts, with lymphocyte percentage having the strongest influence (Figure S11C-D).

When comparing the NTR associations with the ones observed between the extremes in the other populations, we observed a decrease in signal but a high consistency in the direction of associations (Fig. 4). Specifically, 22 positively and 46 negatively associating proteins were in common with the results in RS and LLS-PAROFFS (Fig. 4). These results strengthen the reliability of our previous findings, indicating that the MetaboHealth score is highly informative on the overall health status of individuals, and finally that part of its associations with protein levels in extreme individuals can be explained by genetic pleiotropy, i.e. genetic factors influencing both omics traits.

## Discussion

The MetaboHealth score, along with other ^1^H-NMR metabolomics-based markers, displays risk stratification across a spectrum of health and disease outcomes relevant in ageing research. The score, though trained on mortality, has shown to be an indicator of physiological frailty in middle and older aged individuals. The aim of the current study was a first exploration of molecular pathways that co-vary with the MetaboHealth score. Within this context, our study set out to quantify comprehensive plasma proteome profiles in 150 samples at the extreme ends of the MetaboHealth score distributions from three large Dutch cohorts (LLS-PAROFFS, RS, and NTR, spanning a total dataset of 7,854 individuals). Our findings revealed significant differential expression among 106 plasma proteins and 3 cytokine markers, consistently observed in the RS and LLS-PAROFFS, between the highest (cases) and the lowest (controls) MetaboHealth scores, respectively indicating elevated and reduced health risk. These associations were for most part not confounded by age, sex, BMI, and medication usage, although a part of the proteins associated with the MetaboHealth score contrast could be explained by genetic confounding, as demonstrated analysing discordant MZTs.

The majority of the significant associations (68 out of 106) resulted in negatively associated proteins with the case/control contrast, indicating higher protein levels in samples of healthier subjects (reflected by lower MetaboHealth scores). Functional enrichment of these proteins revealed associations with processes that are often linked to a healthy metabolism, particularly HDL remodeling and cholesterol transport. However, in-depth functional studies using more relevant human tissues, such as liver, would be required to determine the exact biological underpinnings leading to the observed alterations in these pathways. Notably, the most prominent associated proteins were APOA1 and APOA2, crucial components of HDL and widely recognized as protective markers for cardiovascular disease [22, 23]. It should be noted that the associations of APOA1 and APOA2b are likely driven by a shared genetic component with the MetaboHealth score, since these proteins were significantly less discordant in the MetaboHealth score discordant MZ twins. This aspect must be further investigated since a genetic component may be overestimated given that the MetaboHealth score contrast in the (overall younger) MZ twins was rather small in comparison to the cohort studies. Interestingly, the associations with tetranectin (TETN) and gelsolin (GELS) emerged as consistently stable, also in NTR. Both these proteins are under consideration as potential protective markers for various diseases, such as cancer, cardiovascular disease and neurodegeneration [24–26].

We also observed several relevant proteins that were significantly positively associated with the MetaboHealth score contrast. The cytokine markers selected for this study were previously reported to strongly associate with frailty, ageing, age-related disease, and mortality [27–30]. While a large part of the cytokines could not be efficiently detected, which can be attributed to the absence of ongoing infections and acute inflammation at blood sampling, GDF15, IL6, and MIG (gene name = CXCL9) showed significant associations with higher MetaboHealth scores. These cytokines serve different roles in the body and are involved in cell signalling immune response, and inflammation. All three markers are potential biomarkers for aging-related physiological decline and frailty, where IL6 marks chronic inflammation (“inflammaging”) [31–34]; GDF-15 is a senescence associated secretory protein (SASP) and a marker of physiological stress response and mitochondrial dysfunction and MIG is the most prominent marker emerging from the inflammatory aging clock (see materials and Methods) [27]. In this regard, also the 38 significantly positively associated plasma proteins are predominantly involved in inflammatory response, complement and coagulation cascades, and COVID19. CRP, HPT, and LBP, well-known biomarkers assessing the degree of inflammation and immune response, displayed the highest significant associations.

Interestingly the inflammatory component in the positively associated markers also links to COVID19 infection pathways, regardless of their infection status, considering that blood samples used in this study were drawn up to 15 years before the COVID19 pandemic. Noteworthy is that both the proteomics panel and the metabolomics assay composing the MetaboHealth score in plasma, were previously observed to be able to stratify severe cases of COVID19 [7, 20] and clinical outcomes in hospitalized COVID-19 patients[35]. Several plasma proteins previously related to COVID19 were significantly different also in our MetaboHealth score contrast in at least one study. These include the inter-$\alpha$-trypsin inhibitors family, ITIH1, ITIH2, respectively positively and negatively associated, and HRG, LCAT, which are both positively associated to the MetaboHealth score (Figure S9A).

Significant disparities in phenotypic characteristics were observed between cases and controls across the cohorts, mostly in the higher levels of BMI and antihypertensive medication use respectively in LLS-PAROFFS and RS. Interestingly, we observed a significant reduction of lymphocyte percentage consistently among the cases in all studies, although still within normal ranges (20–40%). Concordantly, the systematic adjustment for health and risk factors (age, sex, BMI, lymphocyte and monocyte %, lipid and antihypertensive medication use) resulted in an attenuation of the signal of 89/106 plasma proteins and 3/3 cytokines, with the strongest effect being related to the lymphocyte percentage (Figure S5A-D). A decrease in lymphocyte counts and increased cytokine in blood have previously been associated with a decline in immune system functions that accompanies physiological aging [36–39]. However, although we performed age matching of cases and controls, we did not take into account the heterogeneity in immunological history between individuals, which could have affected our findings[40]. Nevertheless, these observations suggest that the MetaboHealth score successfully identified individuals with an unhealthy metabolic profile given their chronological age. While the correction for cell counts partially accounted for the observed signal, it was evident that hematopoietic variation alone does not explain the observed MetaboHealth score contrast in plasma proteins. Consequently, further investigation is required to elucidate whether the MetaboHealth score and cell count percentages can jointly be more informative in indicating health status of older individuals and secondly what the relation with these parameters and inflammation is.

Conducting heritability analyses within the NTR allowed us to establish that the MetaboHealth score has a heritability of ~ 40%, at least in relatively younger ages (mean age = 36 years). To get a more robust estimate of the heritability of the MetaboHealth score over the lifespan, future studies should ideally make use of larger twin studies that also include individuals of higher ages, such as those present in the Swedish Twin Registry. Nonetheless, the MZT subset provided an ideal setting to further examine the proteomics associations within a genetically identical population. Cytokines did not exhibit significant associations to the MetaboHealth score contrast in the twins, while we found up to 46 negative and 22 positive plasma protein relations. An attenuation of the number and strength of associations indicate that part of the signal may be explained by genetic factors that influence both the MetaboHealth score and quantified proteins. It is essential to highlight that NTR participants had lower MetaboHealth score contrasts as they are younger of age as well as due to the inclusion restriction. Supposedly for the same reasons, diminished associations are also noted when comparing the results in LLS-PAROFFS to RS (Figures S5). A possible explanation for this could be that environmental and/or stochastic factors gain greater importance over genetic influences on the features included in the MetaboHealth score as individuals age, a phenomenon that has previously been observed for epigenetic features and blood-based immune measurements [41–43]. The fact that a large part of the MetaboHealth score seems to be driven by non-genetic factors, future studies should try to determine which lifestyle or pharmacological interventions may be able to improve the score. To this end, combined dietary and physical activity-based interventions in older adults are currently being explored [44, 45]. However, since the score is a reflection of life-long health, the short-term lifestyle interventions are expected to have a limited effect on the score, except in populations with "extremely" high scoring. Furthermore, MetaboHealth score is likely a reflection also of the social economic status, smoking behaviour, and (long-term) dietary habits of an individual which aspects are currently under study.

One aspect of our study that may be regarded as a limitation is that the participants were drawn from population-based cohorts of average ageing volunteers, rather than clinical populations. Consequently, the observed contrast between the extremes of the contrast in MetaboHealth score was relatively modest. This is underscored by the fact that several of the examined cytokines, which are normally used as biomarkers of infection in clinical studies, consistently fell under the detection limit in our studies. We envision the MetaboHealth score to potentially represent a health check in population health management, to provide an indication of the vulnerability of middle aged and older individuals for health professionals such as General Practitioners and in lifestyle centers. A scalable affordable molecular biomarkers indicating overall health may provide a wake-up call in a modifiable health phase, way before onset and diagnosis of specific diseases. The score indeed effectively identified relevant biological profiles within all three populations, indicating that early changes in multiple metabolic and proteome parameters known to reflect decline in health is represented by the score. Given the limited number of individuals in our selection, we do not have the statistical power to explore concrete endpoints such as mortality or frailty in this study, nor were we able to perform any sex-stratified analyses. We consider this study as a proof of principle design to explore the relevance of molecular pathways co-varying with omics scores generated in the ageing field in the context of additional omics levels, to better understand why a score predicts endpoints and which parameters in such pathways might enchange predictive power.

In conclusion, our study confirms MetaboHealth as a robust marker for the inflammatory and metabolic/lipid oriented aspects of aging. In accordance with the current biological aging concept, this score effectively identifies individuals exhibiting reduced lymphocyte counts and increased levels of pro-inflammatory proteins regardless of chronological aging. We believe that this initial investigation supports further integrating the MetaboHealth score with specific proteins such as GDF15 in such a way that measuring the score remains scalable and affordable. These results also warrant further exploration of proteome data combined with metabolomics in cohorts such as UK Biobank to further enhance the prognostic value of the score, increase our comprehension of the aging process and loss of health in individuals identified by the score and the health gain that may be expected by timely intervention.

## Materials and methods

### Study design

This study was conducted starting from the metabolomics data included in the BBMRI-NL consortium originated from the participants to three cohorts: LLS-PAROFFS, RS, and NTR. The LLS-PAROFFS is a population-based cohort with a unique two-generation design, examining 421 Dutch long-lived families [46]. The current work was performed on the first measurements (IOP1) of the second generation, namely the Offspring and their Partners, for a total of 2,313 participants. The RS is a population-based prospective study on individuals living in a specific neighborhood in Rotterdam, prone to cardiovascular endpoints [47]. The current study was set off utilizing the first measurements (RS-I), which included a total of 2,986 participants. The NTR is a prospective study investigating young and adult twins and multiples along with their family members. In this particular study we focused on the MZT and DZT pairs within the cohort, for heritability estimation and implemented a within pairs case–control study design for the 25 most discordant MZT pairs.

Clinical trial number: not applicable.

### Data and sample collection

#### Metabolomics measurements

The metabolomics dataset was generated by the BBMRI-NL Consortium on the EDTA plasma samples of the entire cohorts (LLS-PAROFFS: 2,313 samples, RS: 2,986 samples, and NTR: 2,754 samples). The features were quantified using the high-throughput proton Nuclear Magnetic Resonance (^1^H-NMR) platform made available by Nightingale Health Ltd., Helsinki, Finland. This technique can quantify over 230 metabolic features in a single assay, including lipids, lipoproteins, fatty acid composition, various amino acids, and their derived measures (e.g., ratios) [48, 49]. We employed the dataset quantified in 2014 to ensure the correct projection of the MetaboHealth score model, originally trained on this version of the platform.

### Selection of the participants from the large population studies

#### LLS-PAROFFS and RS

The sample selection was based on the chronological age independent part of the MetaboHealth score, which was obtained as the residual from a linear regression of chronological age on the metabolomics score. The cases were defined as the 25 participants with the highest MetaboHealth score, indicating an unhealthier status, within each cohort separately. Following, to limit the confounding effect of sex and age, the controls were selected as the participants with the lowest score that could have at least one match with the cases in terms of both age, and sex.

#### NTR

The twin population of NTR was composed on 726 complete MZT and 450 complete DZT pairs. We extracted the 25 twin pairs which showed the largest differences in the MetaboHealth score.

### Cytokines quantification

We used previously validated multiplex immunoassays (Luminex platform) to determine plasma protein levels [50]. All assays were performed at the ISO-certified multiplex core facility of the UMC Utrecht, Utrecht, The Netherlands. Before analysis all samples were centrifuged through 0.22 μm spin-X filtration columns (Corning, Corning NY USA) to remove debris. Non-specific (heterophillic) antibodies, which may interfere with the assay, were blocked using Heteroblock (Omega Biologicals, Bozeman, MT, USA) as previously described [10, 11]. If applicable, samples were diluted in high performance elisa buffer (HPE buffer, Sanquin, Amsterdam, the Netherlands). We determined levels of the immunoregulating proteins IL-1β, IL-2, IL-6, IFN-γ, GDF-15, CCL2/MCP-1, CCL3/MIP-1α, CCL5/RANTES, CCL11/Eotaxin, CXCL1/GRO-1α, CXCL9/MIG, PAI-1, BDNF, TRAIL and soluble P-selectin in plasma. These markers were selected based on two criteria, first that they could be measured on our budgetary boundaries using the Luminex platform in the Utrecht University; secondly because they were previously indicated as markers of ageing, mortality, frailty and age-related disease. The last criterion was in great part based on the following studies: A) belonging to the top 15 most informative variables in the iAge clock [27](for the cytokines: CXCL9/MIG, EOTAXIN, CCL3/Mip-1α, IL-1β, IFN-γ, CXCL5/RANTES,CXCL1/GRO-1α, CCL2/MCP1, IL-2; TRAIL, PAI-1); B) recognized as indicators of frailty (P-selectin and BDNF) [28]; C) or marker of chronic inflammation (IL6) [29], and d) finally, widely explored marker for aging, cancer, cardiovascular, and lung disease (GDF-15/MIC-1) [30].

Although the majority of the features (73%) were quantified in undiluted samples, PAI1 was measured with a dilution rate of 1/10, and, GDF15, RANTES, and BDNF, with a dilution rate of 1/100.

### Protein digestion

Protein digestion was performed on an Agilent Bravo liquid handling Platform following Vollmy et. al. [20]. A pooled QC sample was prepared by pooling an equal amount of each digested sample. Then all samples were diluted 40 times with TFA 1% to bring them to an approximate concentration of 10 ng/uL. Finally, the samples were loaded onto Evotips (Odense, Denmark) using an Agilent Bravo liquid handling platform.

### LC–MS data acquisition

The digested samples were measured using the 60 SPD method of a Evosep One (Odense, Denmark) on a EV-1109 column (C18, 8 cm × 150 µm, 1.5 µm, Evosep, Denmark) coupled to a timsTOF-HT (Bruker, Germany) equipped with a Captive Spray source and operating in DIA-PASEF adapted from Skowronek et. al. [51] Briefly, two ion mobility windows per dia-PASEF scan with 12 variable isolation window widths adjusted to the precursor densities were used. The ion mobility range was set to 0.6 and 1.5 cm^−1^. The accumulation and ramp times were specified as 100 ms for all experiments. Source capillary voltage and temperature were set to 1800 V and 180 °C. Drying and sheath gas were set to 3 L/min. The pooled QC samples were injected every 8 samples.

### Processing of proteomics data

Raw data were processed using DIA-NN 1.8.1 [52], Peptides were searched against an in-silico predicted library computed from the human proteome with isoforms (UniProtKB and TrEMBL, 103,830 protein entries and 20,560 genes) and the common protein contaminants with 2 missed-cleavages and no variable modification. Match Between Run was used and the heuristic inference was disabled. MS1 and MS/MS mass accuracy were set to 10 and 20 ppm respectively.

The protein intensities were computed using the maxLFQ algorithm implemented in the DIA-NN R-package. For this only the prototypic precursors that satisfy the following criteria were considered: Q.value ≤ 1%, missing value ≤ 20% and RSD ≤ 40% in the QC samples. The protein groups were filtered at lib.Q.Value ≤ 1%, lib.PG.Q.value ≤ 1%.

### Covariates

Data on age (in years), sex (males/females), BMI (kg/m^2^), cell counts (%), lipid medication, and blood pressure lowering medication were reported within the BBMRI-nl Consortium. These covariates were evaluated as possible confounders as they are known to be associated with both the metabolomics and the proteomics datasets. Age and sex were self-reported, and BMI was calculated based on weight and height. The cell counts percentage was defined as the measured monocytes and lymphocytes percentage, taking the granulocyte percentage as given.

## Statistical analyses

### Preprocessing

#### Metabolomics and MetaboHealth score projection

We applied the same quality control previously described by Deelen et al. [11], using the R package MiMIR [53]. While the Nightingale Health platform measures over 250 metabolomics features, we focused our attention on the 14 metabolomics variables included in the MetaboHealth model (list of analytes can be found in the Supplementary Table S1). Then, we applied a logarithm transformation to the analytes, while adding a value of 1 to all analytes containing any zero as a value. Afterwards a z-scale normalization was applied separately within each cohort to minimize batch effects. Finally, we projected the MetaboHealth score using the coefficients indicated by Deelen et al. [11, 53].

#### Cytokines

Initially, we assessed the occurrence of samples reported as under the lower detection thresholds, comparing cases and controls, separately for each feature. Subsequently, we employed the Fisher test to determine the statistical significance of these difference (*p*-value $\le$ 0.05). Following, we proceeded to exclude six out of fifteen features that consistently fell below the lower detection thresholds in most of the quantified samples (namely, IL2, TRAIL, GRO1a, IFNg, IL1b, and PAI1 were undetected in more than 65% of the samples). There were no values recognized as outlier samples, considered as values more than 5 standard deviations (SD) away from the mean of the feature. Finally, the remaining nine features were first log transformed and then z scaled separately per biobank to reduce batch effects.

#### Proteomics

We applied a similar approach to the proteomics. We evaluated the differential patterns in missing values of the features between the cases and controls. Features with more than 5% missing values (20 missing) were subsequently excluded, resulting in a set of 261 analytes, from the 337 initial set 8 values (0.03%) were recognized as outliers, meaning that they resulted as the values 5 SD away from their mean of the feature and set as missing information. We imputed the remaining 114 missing values (0.4%) using the non-linear iterative partial least squares method (nipals), implemented in the R package *pcaMethods*. Finally, to enhance comparability and facilitate downstream analysis we performed a log transformation and a z-scaling of the features per biobank.

### Linear association and meta-analyses in RS and LLS-PAROFFS

Initially, we applied linear regression models across the entire dataset to assess the associations between each cytokines and proteomics features separately, with the case/control status of the participants. These analyses accounted for potential confounding factors (age, sex, BMI, cell counts, and usage of lipid medication and blood pressure lowering medication). Subsequently, we attempted to evaluate the associations independently for each cohort (LLS-PAROFFS and RS) and conducted a meta-analysis. The meta-analysis was performed with a restricted maximum likelihood estimator using the package *metafor* in R. To correct for multiple testing, we applied False Discovery Rate (FDR). Nominal *p*-values are reported in the main text for clarity; all findings were verified against FDR thresholds.

### Enrichment analyses and network analysis

We performed the functional enrichment of the most interesting proteomics features using the web-tool *Enrichr* [54]. We evaluated the Gene Ontology (GO) Biological Processes (BP), the KEGG pathways, and Reactome. Firstly, we performed an enrichment analyses on the full set of 337 proteomics features to evaluate the functions overall characterizing to the proteomics platform (Figure S8A-B). Secondly, we analyzed separately the 38 positively and the 68 negatively associated proteins to the MetaboHealth’s extremes corrected for sex, age, and BMI (Figure S8C-H). To ensure a fair enrichment analysis of the significantly associated proteins we used the full list of 337 proteins as the background of possible analytes. To better interpret at the GO BP results we used the R package *rrvgo* (threshold = 0.7), which is able to summarize the redundant information in the database [55]. We used FDR to correct for multiple testing.

### Analyses in the Netherlands Twin Register

First, we examined heritability of the MetaboHealth score within Netherlands Twin Register. MZTs share their (almost) complete DNA sequence, while DZTs share on average 50% of their segregating genes. Any differences in correlations between MZTs and between DZTs offers a first hint on genetic influences in the signal. We obtained an estimate of the heritability of a trait (h^2^) using the formula: h^2^ = 2(r_MZTs_-r_DZTs_), where r denotes the correlation between the twins [21].

Secondly, we assessed the associations between the cytokines and serum protein features with the case/control status of the selected MZTs with the highest MetaboHealth differences. To do this we used linear mixed models to consider the family status, while allowing to systematically correct for potential covariates. Finally, FDR to correct for multiple testing.

### Ethics statements

#### LLS-PAROFFS

The LLS-PAROFFS protocol was approved by the Medical Ethical Committee of the Leiden University Medical Center before the start of the study (P01.113). In accordance with the Declaration of Helsinki, the LLS-PAROFFS obtained informed consent from all participants prior to their entering the study.

#### RS

The RS protocol was approved by the Medical Ethics Committee of the Erasmus MC Rotterdam, the Netherlands. (*MEC 02.1015*) and by the Dutch Ministry of Health, Welfare, and Sport (Population Screening Act WBO, license number 1071272–159521-PG). In accordance with the Declaration of Helsinki, the RS obtained written informed consent from all participants prior to their entering the study.

#### NTR

The NTR study protocol was approved by the Central Ethics Committee on Research Involving Human Subjects of the VU University Medical Centre, Amsterdam, an Institutional Review Board certified by the U.S. Office of Human Research Protections (IRB number IRB00002991 under Federal-wide Assurance- FWA00017598. In accordance with the Declaration of Helsinki, the NTR obtained informed consent from all participants prior to their entering the study.

### Data sharing

LC–MS data have been deposited to the ProteomeXchange Consortium via the PRIDE partner repository with the dataset identifier PXD057946. Phenotypic characteristics, cytokine levels, and metabolomics quantifications are available upon reasonable request at BBMRI-nl https://www.bbmri.nl/services/samples-images-data.

## Supplementary Information

Supplementary Material 1.

Supplementary Material 2.

Supplementary Material 3.

## Data Availability

Mass Spectrometry data have been deposited to the ProteomeXchange Consortium via the PRIDE partner repository with the dataset identifier PXD057946. Phenotypics characteristics, cytokine levels, and metabolomics quantifications are available upon reasonable request at BBMRI-nl https://www.bbmri.nl/services/samples-images-data.
