# Supplementary material for: Extreme MetaboHealth scores in three cohort studies associate with plasma protein markers for inflammation and cholesterol transport
Source: Immun Ageing. 2025 Sep 9;22:34. doi: 10.1186/s12979-025-00527-7 (PMC12418662; doi:10.1186/s12979-025-00527-7)

Figure S1: MetaboHealth-driven selection and main phenotypic differences

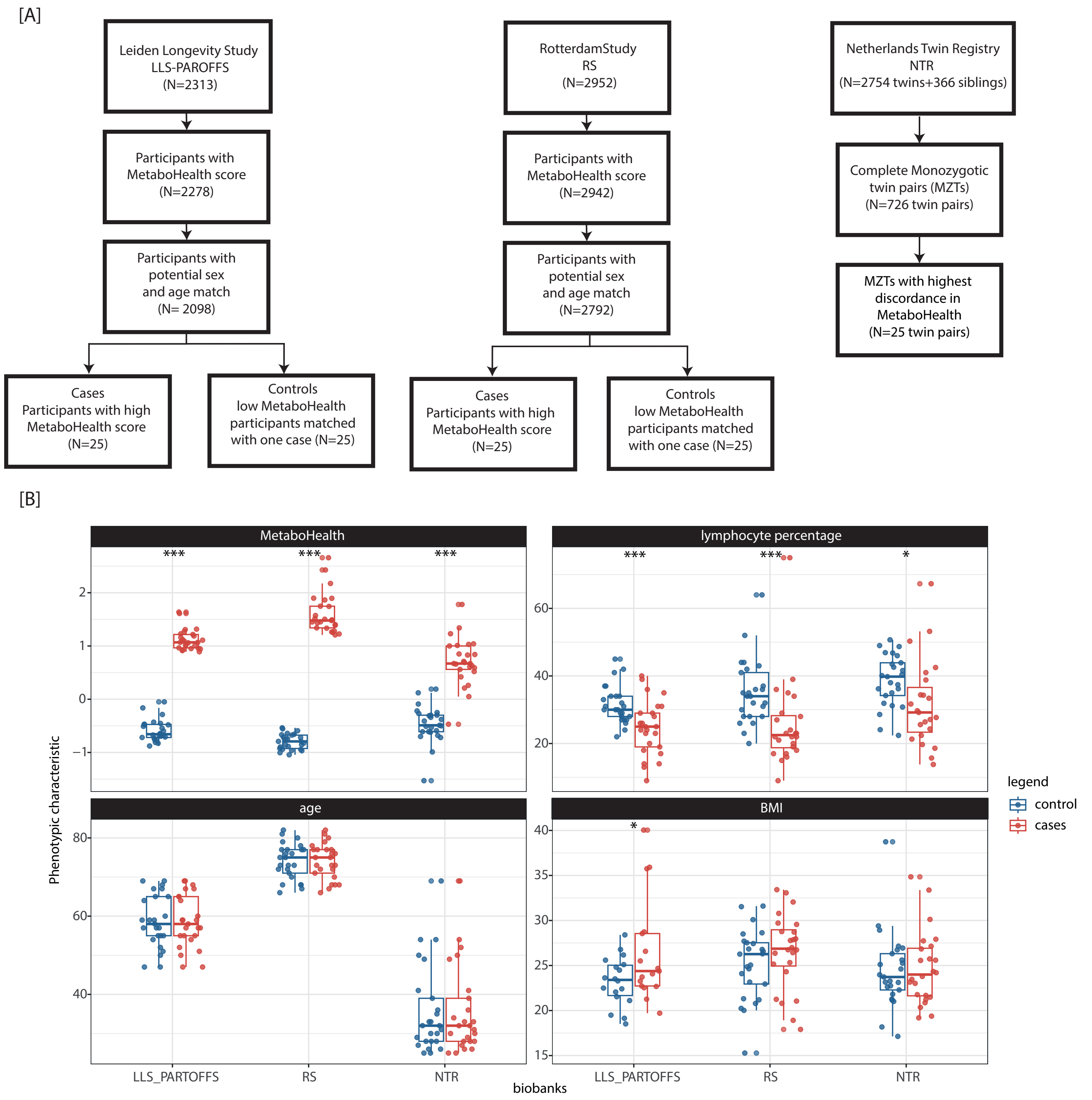

Figure S2: Quality control of the cytokines in LLS-PAROFFS and RS.

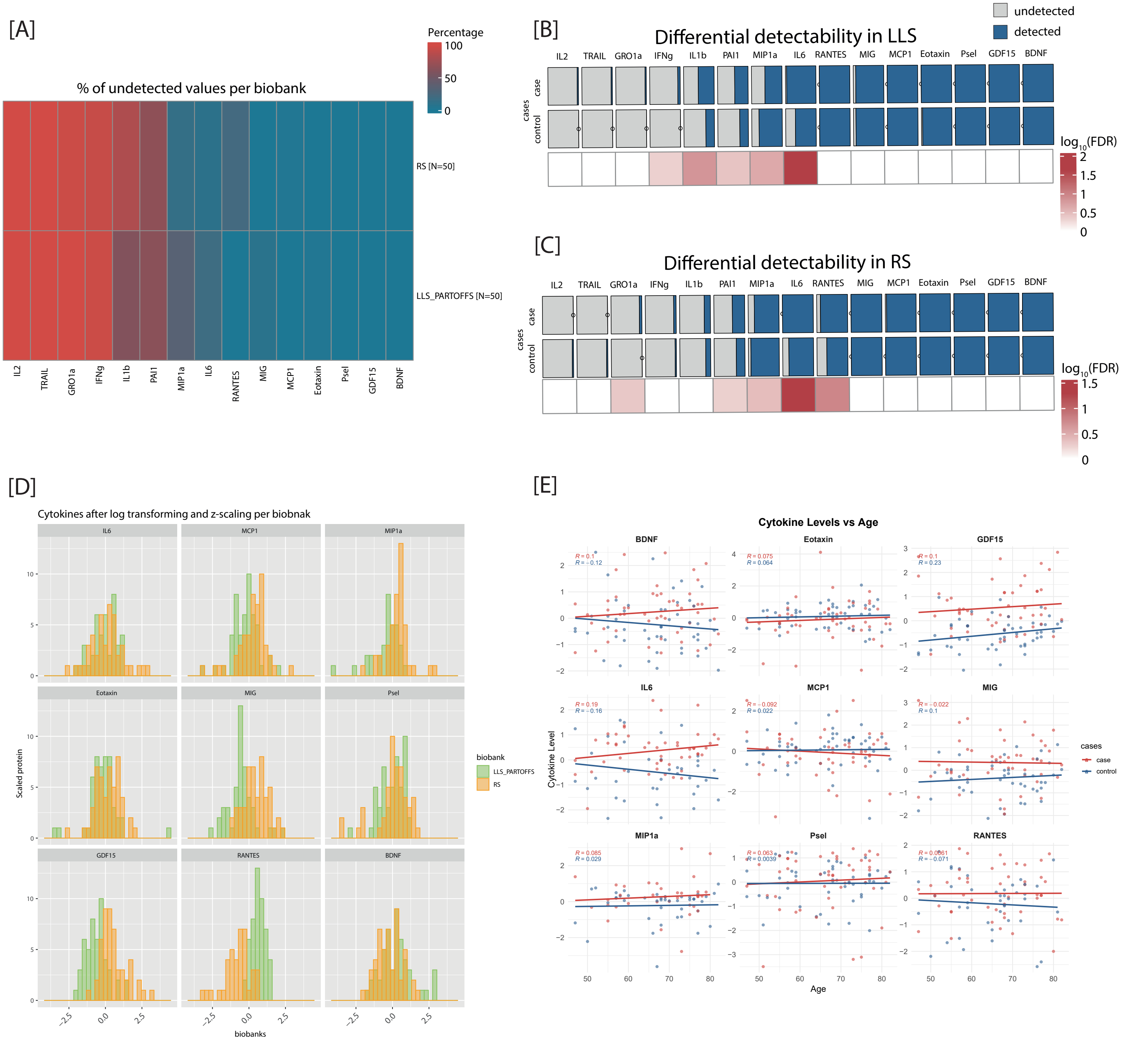

Figure S3: Sensitivity analysis and Meta-analysis of the cytokine's associations with the MetaboHealth extremes

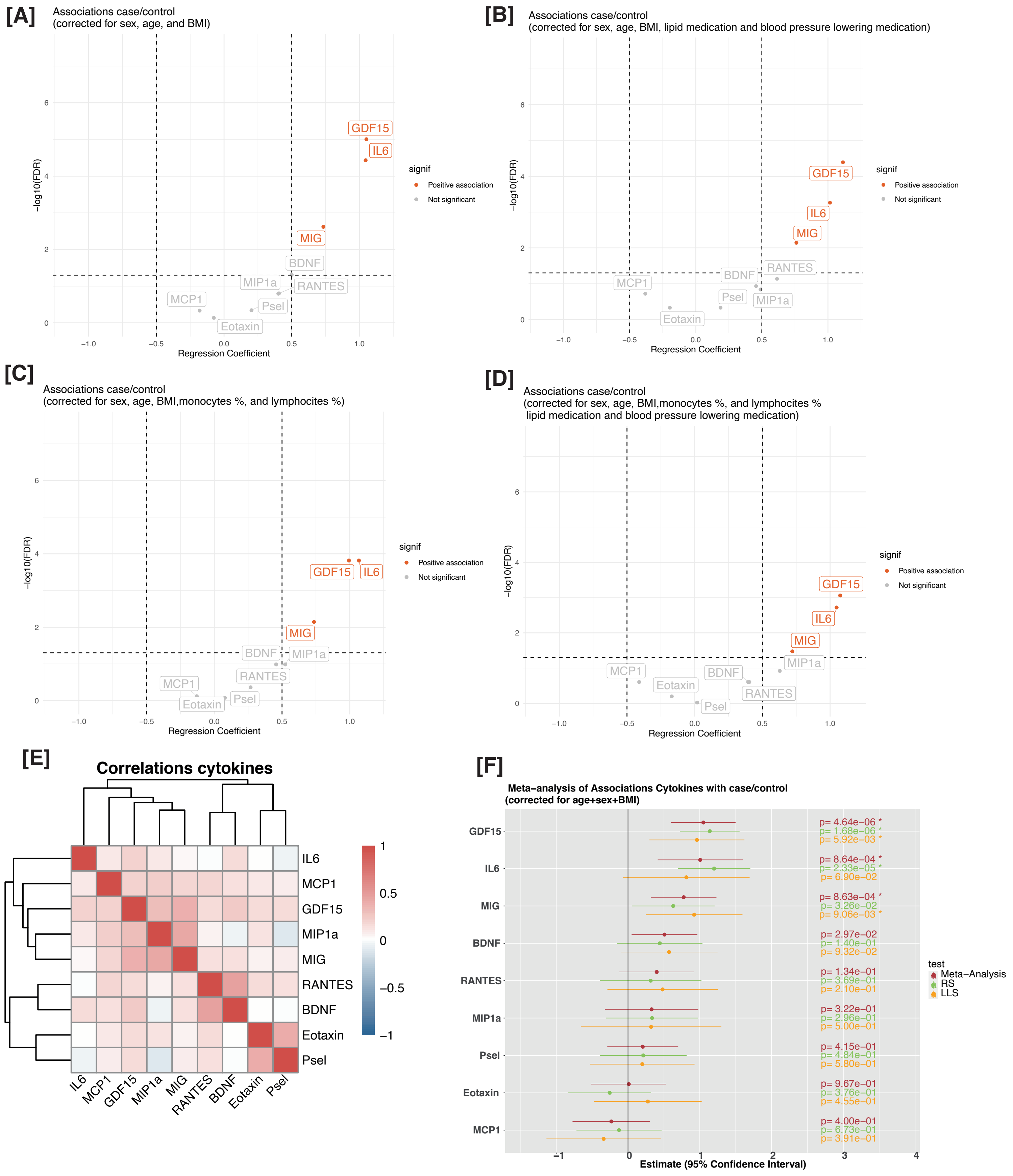

Figure S4: Quality control of the proteomics dataset

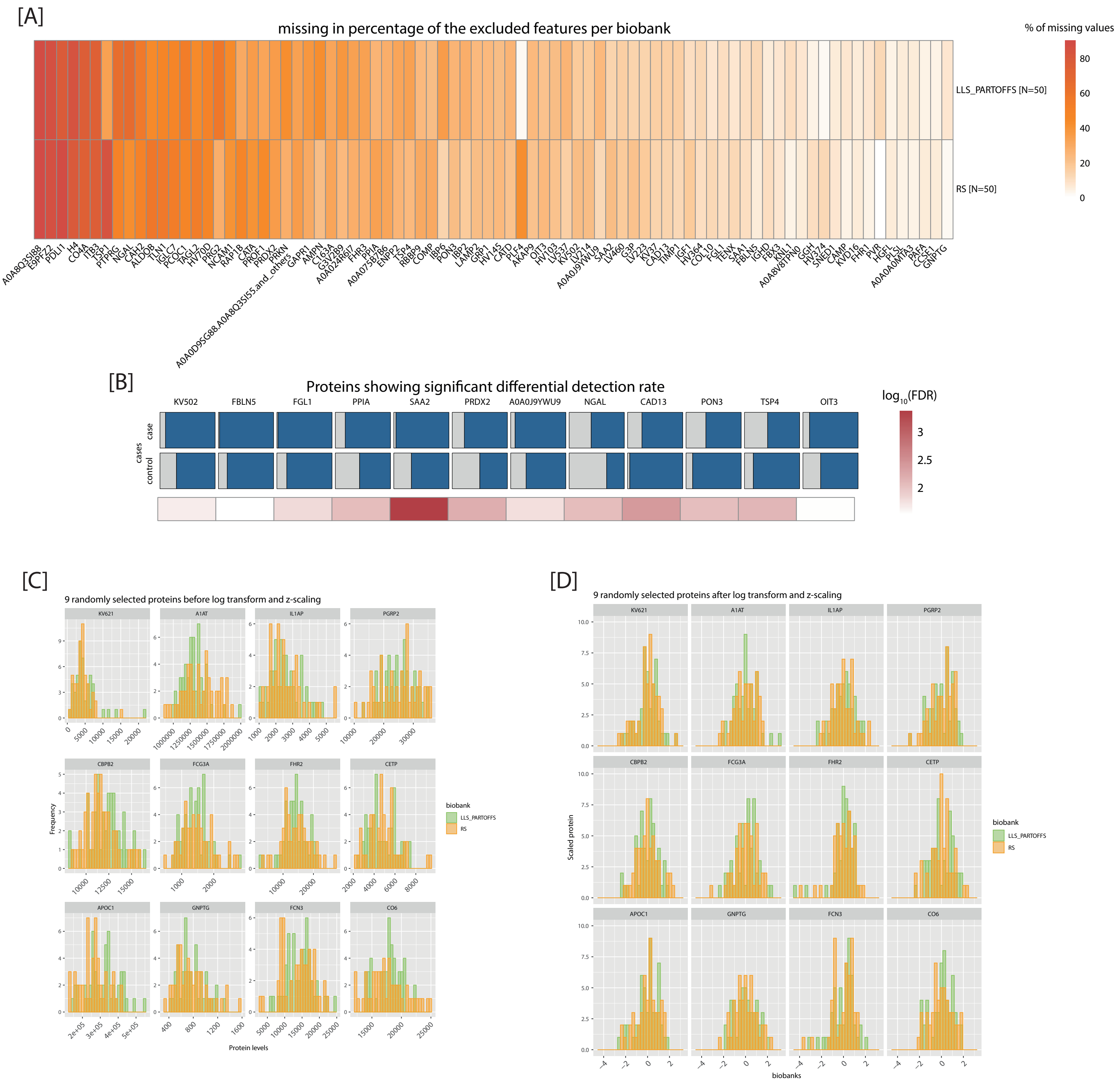

Figure S5: Sensitivity analyses and Meta-analyses of the plasma protein with MetaboHealth extremes in RS and LLS-PAROFFS

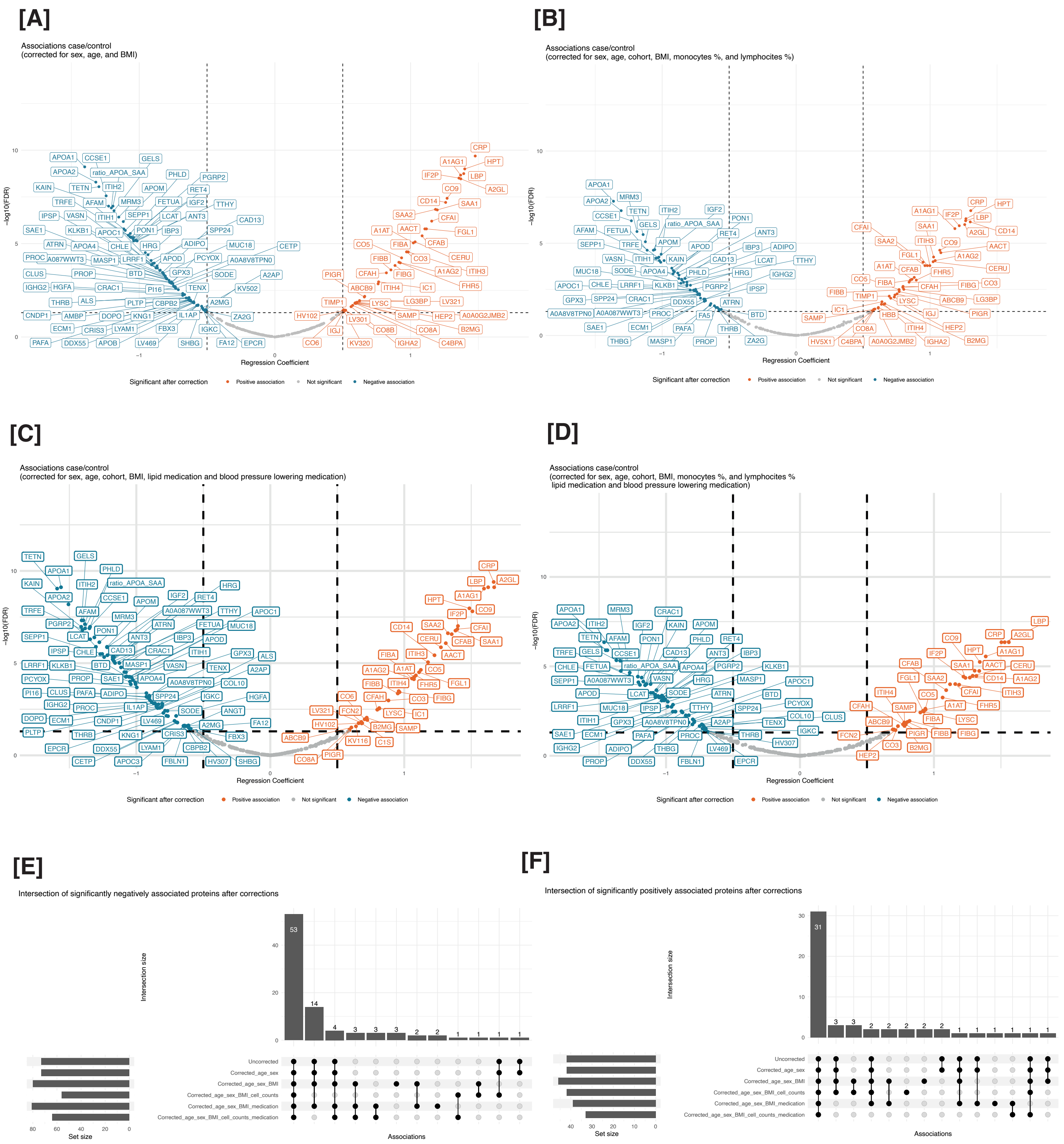

Figure S6: Meta-analyses of the associations between the proteomics features and the MetaboHealth's extremes

[A]

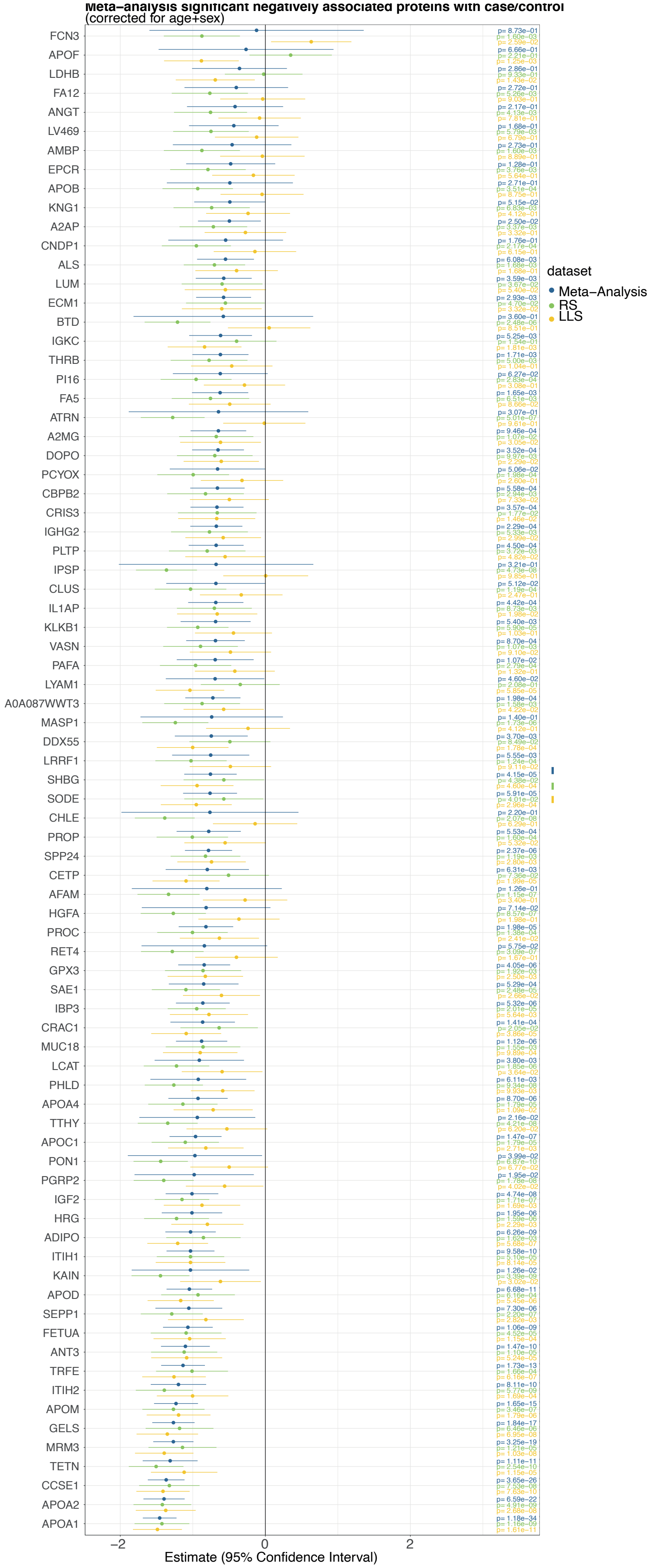

[B]

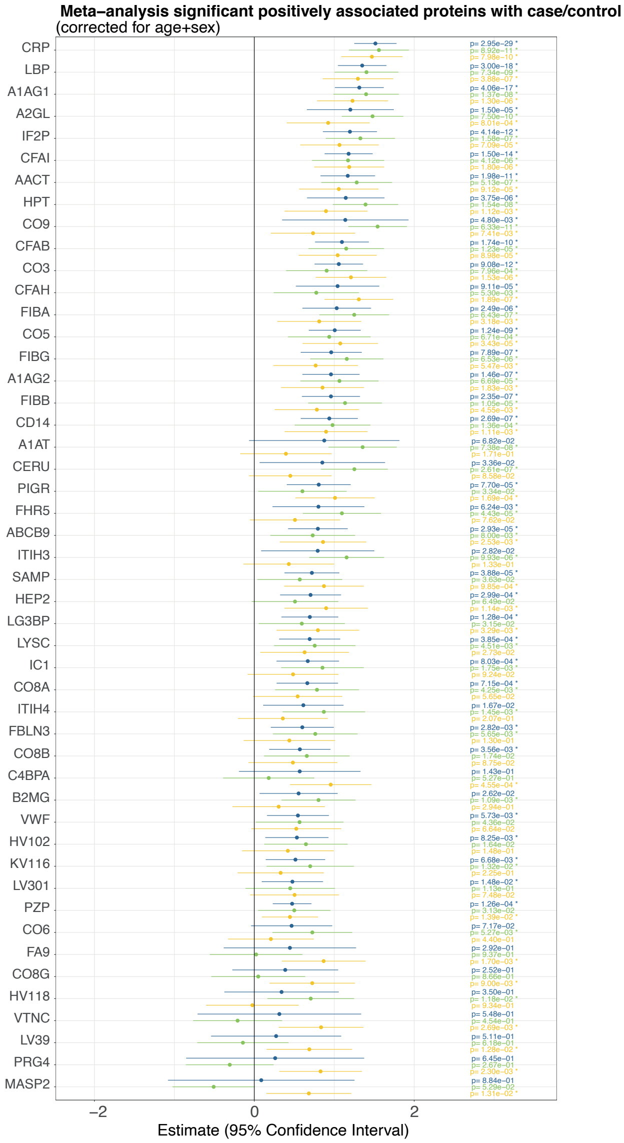

Figure S7: Correlations of the significantly associated proteins

[A]

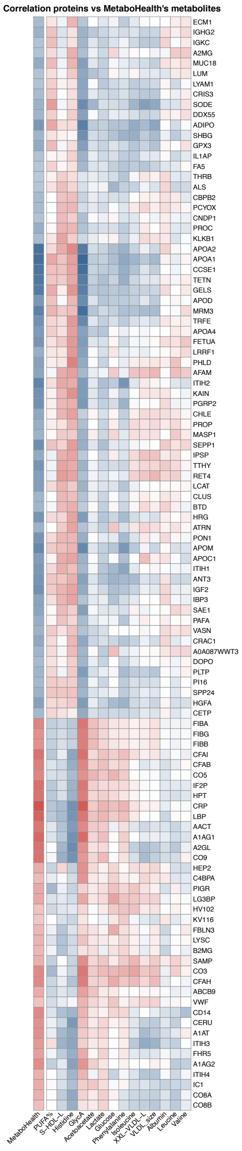

[B]

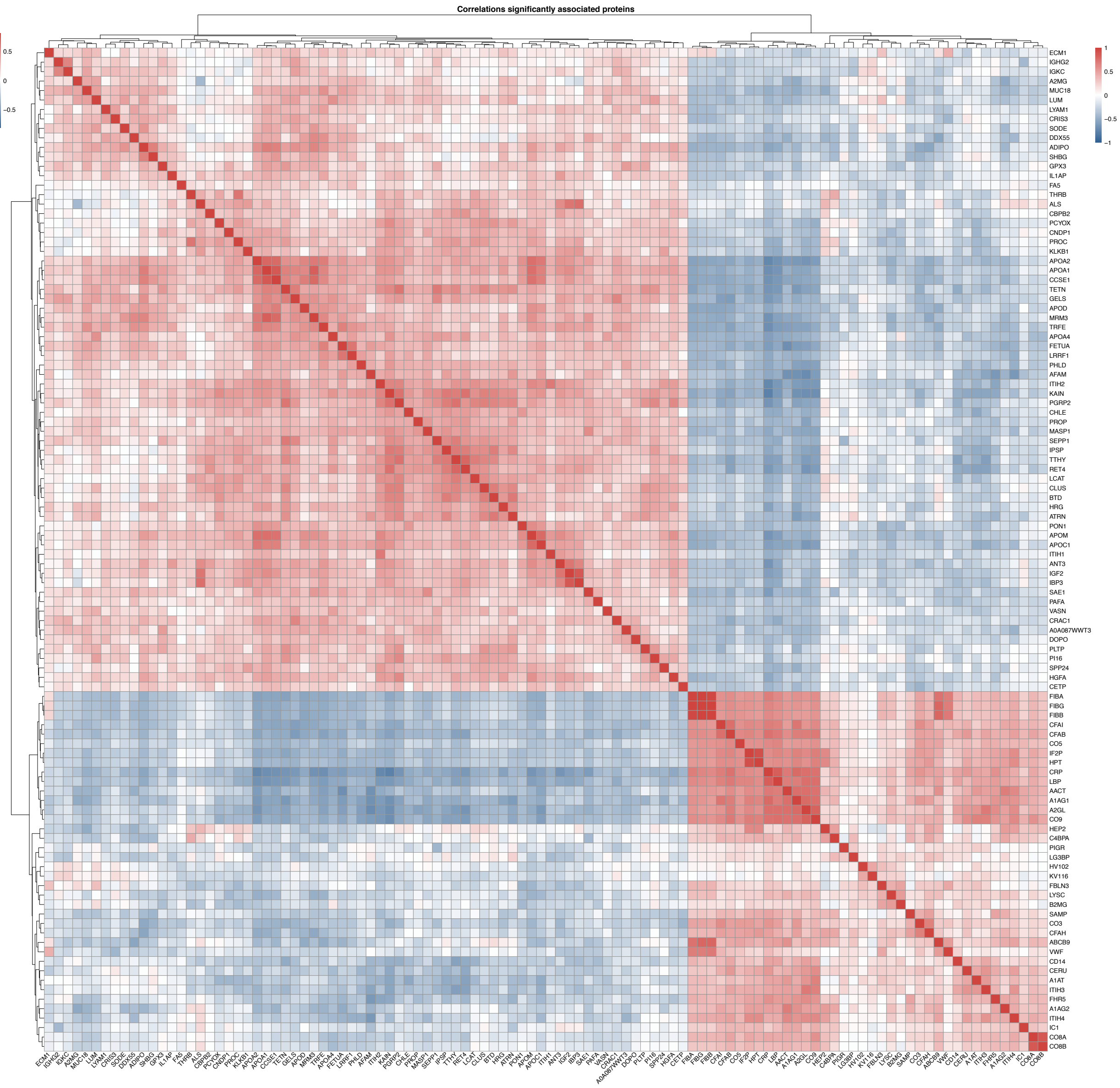

Figure S8: Functional enrichments in the proteomics dataset

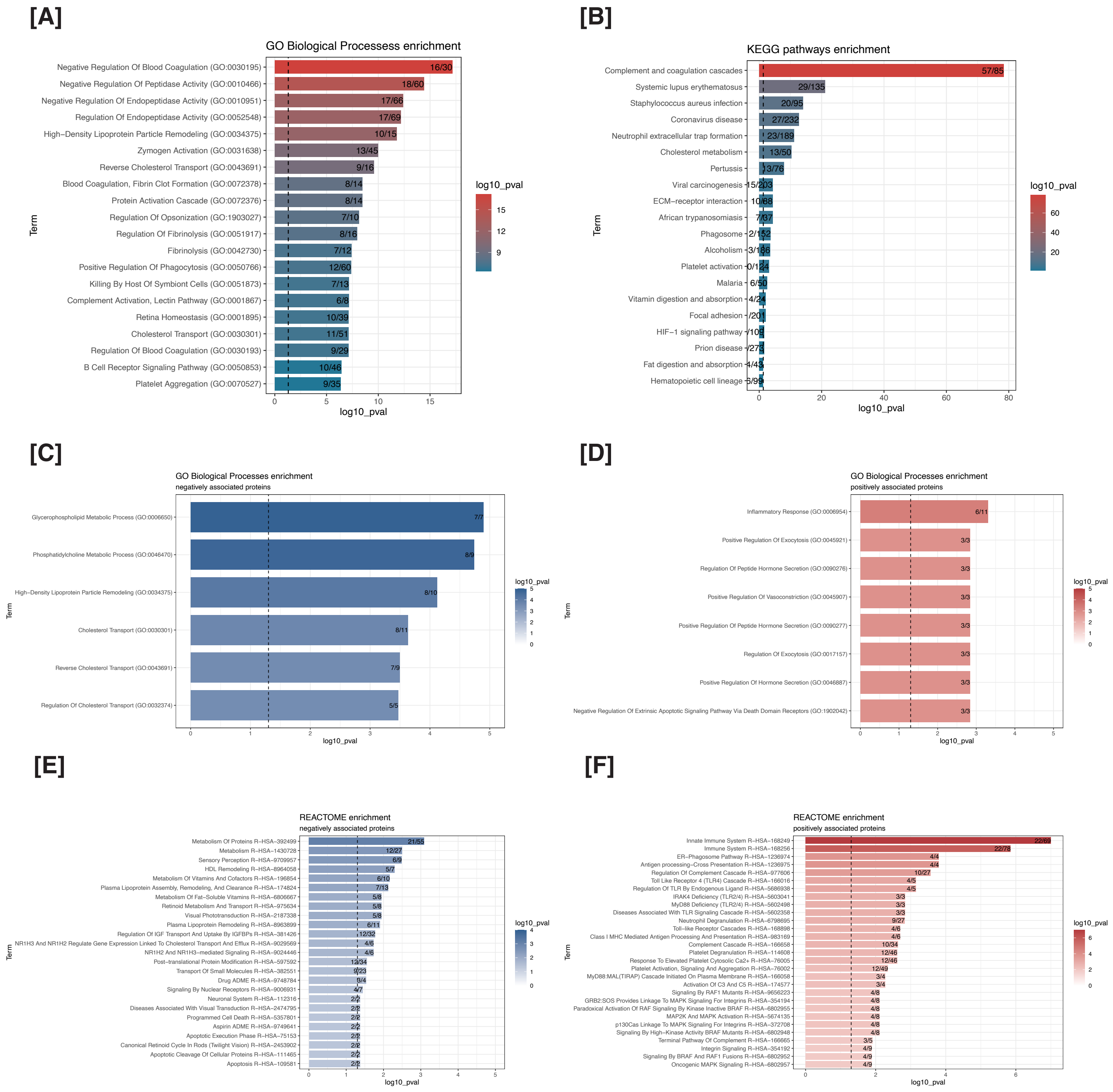

Figure S9: Covid19 related proteins show differences with MetaboHealth

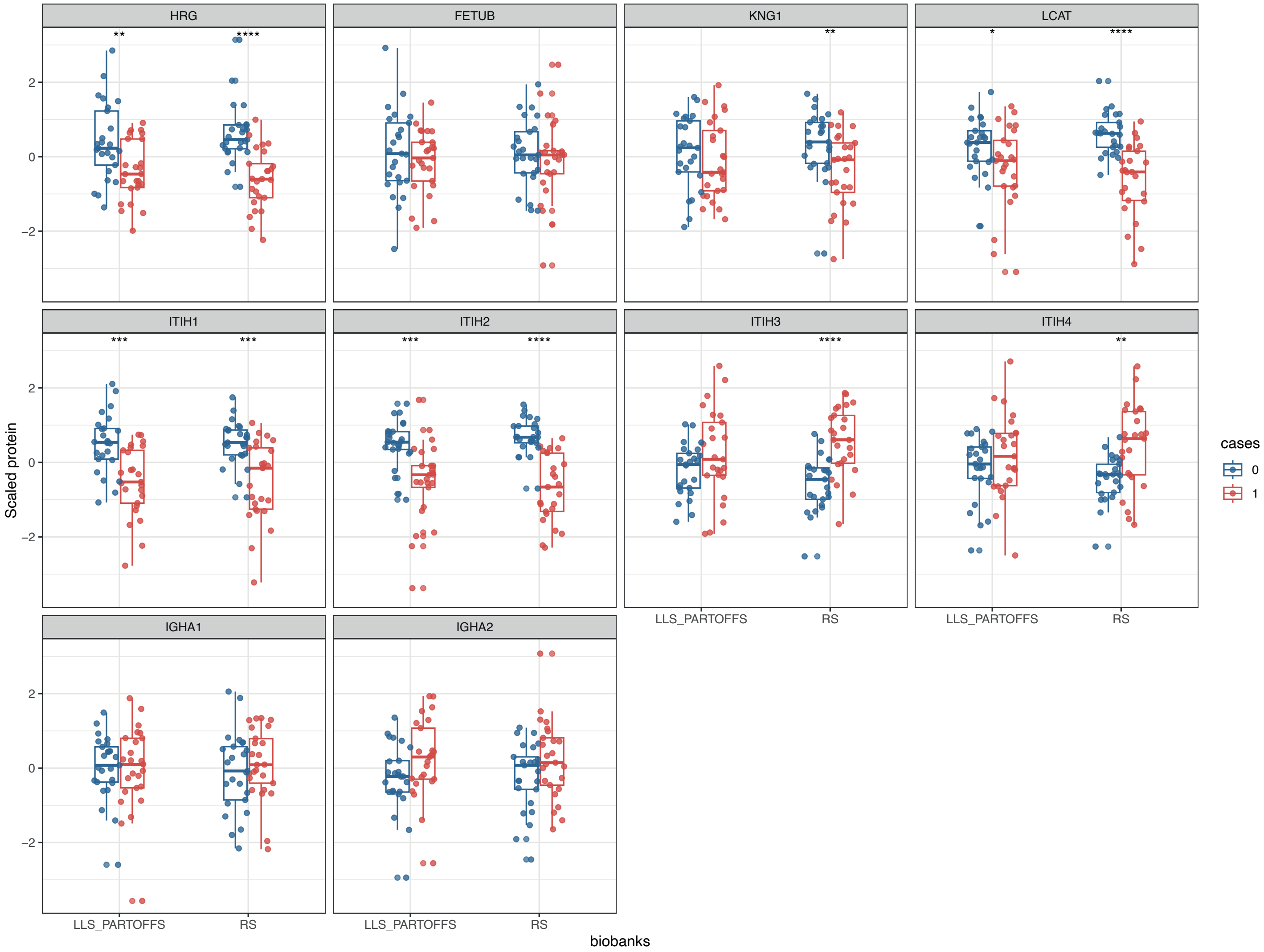

Figure S11: Associations in NTR

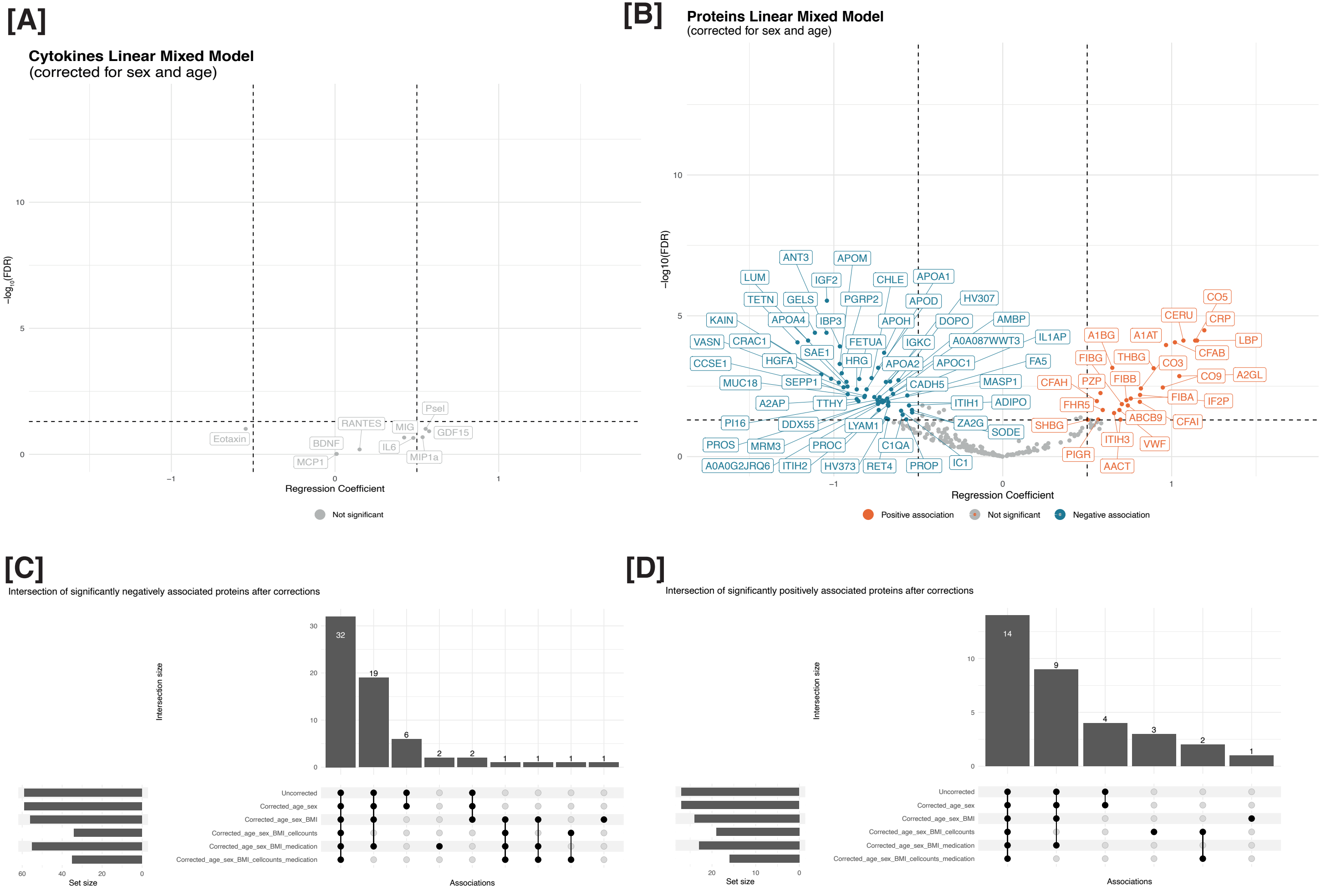

Figure S12: Complete heatmap profile of the significant proteins

[A]

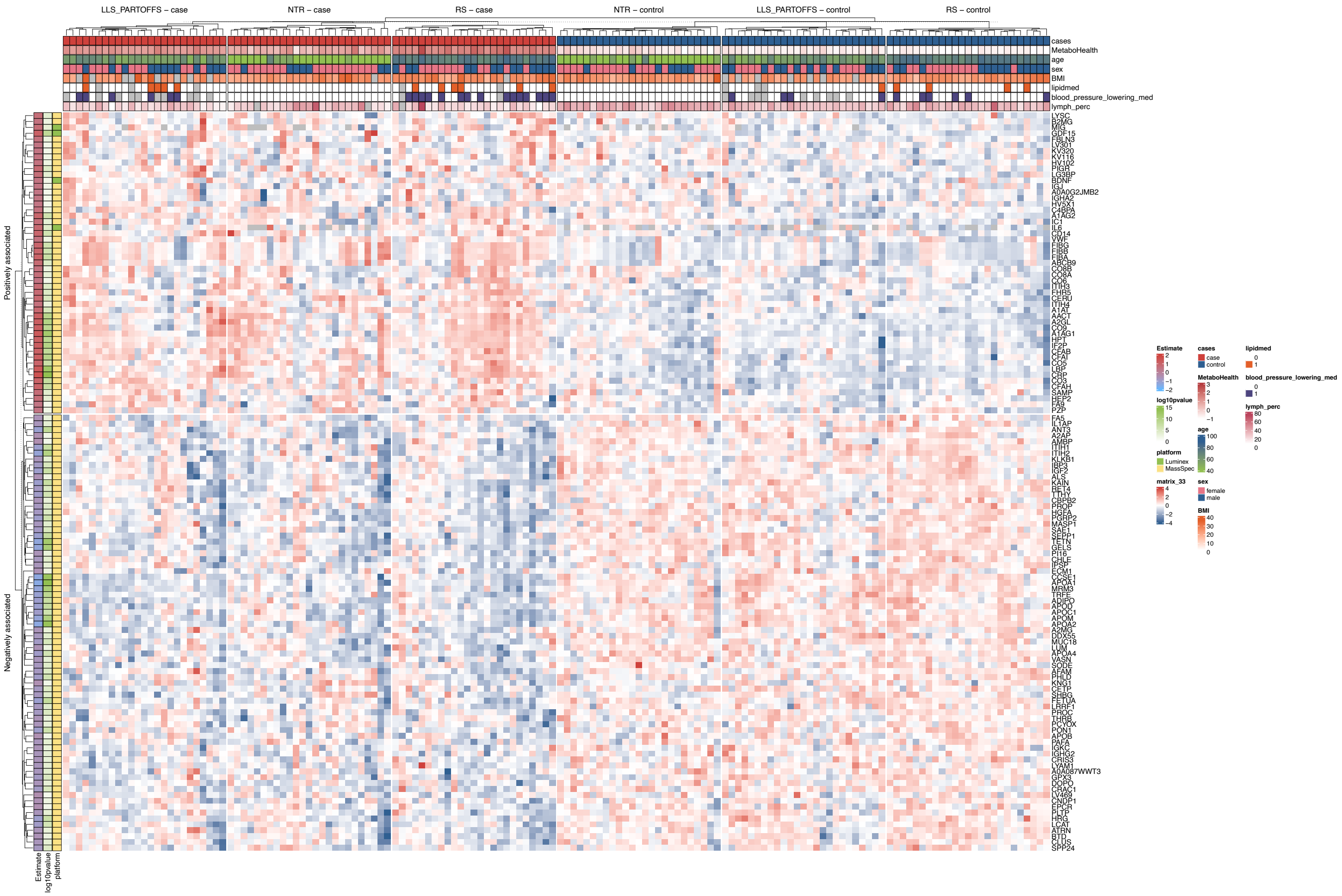

Supplement: Supplementary file 1 — Supplementary Material 1. [file 12979_2025_527_MOESM1_ESM.pdf]
